# The WHO guideline on drugs to prevent COVID-19: small numbers- big conclusions

**DOI:** 10.12688/wellcomeopenres.16741.1

**Published:** 2021-03-30

**Authors:** William HK Schilling, James J. Callery, Arjun Chandna, Raph L Hamers, James A Watson, Nicholas J White

**Affiliations:** 1 Centre for Tropical Medicine & Global Health, Nuffield Department of Medicine, University of Oxford, Oxford, OX3 7BN, UK; 2 Mahidol Oxford Tropical Medicine Research Unit (MORU), Faculty of Tropical Medicine, Mahidol University, Bangkok, Thailand; 3 Cambodia Oxford Medical Research Unit, Angkor Hospital for Children, Siem Reap, Cambodia; 4 Eijkman Oxford Clinical Research Unit, Eijkman Institute for Molecular Biology, Jakarta, Indonesia

**Keywords:** SARS-CoV-2, COVID-19, Coronavirus, Prophylaxis, Pre-exposure, Guideline

## Abstract

The World Health Organization living guideline on drugs to prevent COVID-19 has recently advised that ongoing trials evaluating hydroxychloroquine in chemoprophylaxis should stop. The WHO guideline cites “high certainty” evidence from randomised controlled trials (RCTs) that hydroxychloroquine prophylaxis does not reduce mortality and does not reduce hospital admission, and “moderate certainty” evidence of poor tolerability because of a significantly increased rate of adverse events leading to drug discontinuation. Yet there is no such evidence. In the three pre-exposure chemoprophylaxis RCTs evaluated in the guideline there were no deaths and only two COVID-19-related hospital admissions, and there was a mistake in the analysis of the number of discontinuations (after correction there is no longer a statistically significant difference between those taking the drug and the controls). Guidelines on the prevention and treatment of COVID-19 should be based on sufficient verified evidence, understanding of the disease process, sound statistical analysis and interpretation, and an appreciation of global needs.

## Disclaimer

The views expressed in this article are those of the author(s). Publication in Wellcome Open Research does not imply endorsement by Wellcome.

## Introduction

On 2 March 2021 the World Health Organization (WHO) issued a guideline on COVID-19 prophylaxis which included unusual judgements with far reaching implications derived from small numbers of observations ^1, 2^. There are several analogies between the legal system and clinical investigations, and in particular the interpretation of clinical trials and the production of treatment guidelines. Both seek the truth, both review the strengths and weaknesses of evidence, and both end in a judgement. The WHO guideline uses standard methodological approaches to evaluate and grade clinical research outputs and to generate guidelines ^3^. In general, this is a conservative process requiring a substantial quality evidence base for definitive recommendations. This reduces uncertainty in the assessments, and it helps ensure that the consequent guidelines are robust. We argue that this has not happened for the WHO guideline on drugs to prevent COVID-19.

The WHO guideline provides a strong recommendation against hydroxychloroquine in COVID-19 pre- or post-exposure prophylaxis ^1, 2^. In many ways, hydroxychloroquine has become the COVID-19 pariah, but it still deserves a fair trial. It is true that hydroxychloroquine for both treatment and prevention was intensely politicised, and it was recommended prematurely by many governments, institutions and prominent individuals early in the COVID-19 pandemic. It should not have been. It is also true that definitive large randomised controlled trials (RCTs) have shown unequivocally that hydroxychloroquine is not life-saving in hospitalised patients ^4^. This is the stage of the disease when anti-inflammatory drugs, such as dexamethasone or interleukin 6 (IL-6) receptor antagonists, but not antiviral drugs (notably remdesivir), have proved lifesaving ^5, 6^. However, in contrast to the large randomised controlled trials (RCTs) in hospitalised patients, relatively few patients have been enrolled in studies of hydroxychloroquine in early treatment, or in post-exposure (PEP) or pre-exposure prophylaxis (PrEP). In the three published, or posted, pre-exposure prophylaxis RCTs there were very few endpoints ^1, 2^. Nevertheless, the WHO guideline has concluded definitively that hydroxychloroquine does not provide useful benefit in any of these situations. The WHO guideline group has also taken the unusual step of advising funders and researchers that they should reconsider the initiation and continuation of ongoing trials, i.e. they should stop. So, although it is described as a “ *living guideline*”, without further evidence it is unlikely that this particular guideline will live much longer. Case closed.

From a statistical perspective both the justice system, and the institutions which issue disease prevention and treatment regulatory approvals and guidelines, focus primarily on demonstrating proof beyond reasonable doubt. Trials that lead to conviction have proved guilt beyond reasonable doubt. Pre-registration RCTs aim to prove efficacy of a drug beyond reasonable doubt, in addition to showing that the cost of this efficacy is not too high in terms of tolerability and safety. In the WHO COVID-19 prophylaxis guideline the opposite is being done. A definitive statement about *lack* of clinical utility is being made. According to the guideline the highest efficacy estimate compatible with the data (lower end of the confidence interval) is not a clinically useful effect. Despite the small number of endpoints, the guideline claims that hydroxychloroquine, taken for prevention, results in no important differences in mortality, admission to hospital, or laboratory confirmed COVID-19 ^1, 2^. It also claims that adverse events (AEs) leading to drug discontinuation is a significant problem for hydroxychloroquine prophylaxis. Both contributed to the WHO judgement that hydroxychloroquine should not be used, and should not be evaluated further in COVID-19 prophylaxis trials ^1, 2^.

There is certainly not enough evidence to recommend hydroxychloroquine for COVID-19 prophylaxis (there never has been), but is this small and heterogeneous evidence base enough to state conclusively, as the WHO guideline has done, that hydroxychloroquine does not provide a modest but worthwhile benefit? Does it justify the implicit recommendation that ongoing RCTs in the prevention of COVID-19 should stop now? Nearly all the evidence used to generate this strong recommendation has been in the public domain for several months. It comprises three RCTs in post-exposure prophylaxis (PEP-which is close to early treatment) and three in pre-exposure prophylaxis (PrEP- true prevention) ^1, 2^. Two studies used confirmed or suspected COVID-19 (mainly suspected) as their primary endpoints, and the other four used laboratory-confirmed COVID-19. Dosages differed – notably, the largest PrEP study (>75% of the PrEP data) used a much lower hydroxychloroquine dose, closer to that used in antimalarial chemoprophylaxis rather than the more widely used rheumatoid arthritis doses used in other COVID-19 prophylaxis trials ^7^. Another PrEP study had only a single case of COVID-19 ^8^; and none of the data in any of the included studies were collected outside of North America or Europe. There were other differences which overall may be summarised as “substantial heterogeneity” between studies.

**Figure 1.**
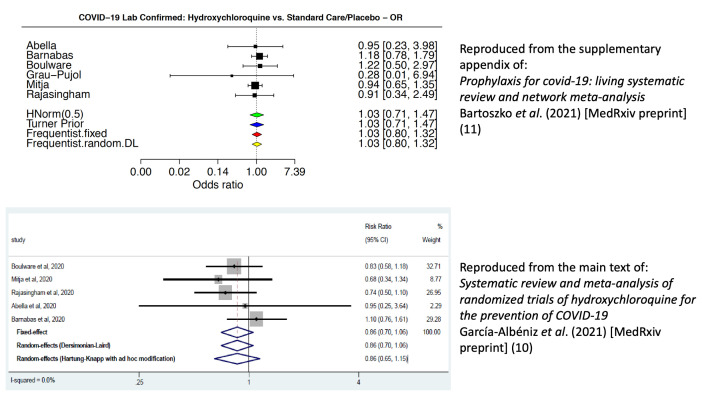
Comparison of forest plots for the effect of hydroxychloroquine in the prevention of COVID-19. The top forest plot (treatment effect summarised as odds ratios) is reproduced from 11 (supplementary materials) under a CC-BY-NC 4.0 International license; the bottom forest plot (treatment effects summarised as risk ratios) is reproduced with permission from 10 (main text) under a CC-BY-NC-ND 4.0 International license. Differences in estimated effects reflect differences between endpoint definitions and the use of intra-study adjusted treatment effects.

These six randomised controlled comparisons enrolled 6,059 participants, but they generated relatively few endpoints (suspected or confirmed COVID-19, hospital admission or death). In the three PrEP trials there were only 26 confirmed COVID-19 cases in total (15 out of 1,197 randomised to hydroxychloroquine, 11 out of 687 randomised to placebo). With so few events and considerable heterogeneity in design, the meta-analysis is sensitive to the methods employed. A previous meta-analysis chose to use the appropriately adjusted primary endpoints reported in each hydroxychloroquine prevention study (e.g. one study was a cluster randomised trial ^9^ so adjustment for cluster was necessary) ^10^. This estimated a meta-analytic risk ratio of 0.86 (95% confidence interval [C.I.] 0.70 to 1.06) in favour of hydroxychloroquine. In comparison, the WHO guideline used laboratory confirmed COVID-19 (asymptomatic and symptomatic) for the primary endpoint in their meta-analysis of virological effect, without intra-study adjustments, resulting in a meta-analytic odds ratio of 1.03 (95% C.I. 0.80 to 1.32) ^1, 2^. Figure 1 compares the two analyses.

The WHO guideline development panel decided that “ *Mortality would be the outcome most important to individuals, followed by need for hospital admission, laboratory confirmed SARS-CoV-2 infection, and adverse effects leading to discontinuation*” ^1, 2^. The review determined that there was no important difference in mortality, admission to hospital, or laboratory confirmed COVID-19, and that the evidence quality to support these statements was high. The four-star “ *High GRADE rating*” is defined as: “ *the authors have a lot of confidence that the true effect is similar to the estimated effect*”. Yet there were only 13 deaths in total in the six prophylaxis trials, and they were all from one cluster-randomised non-blinded PEP trial ^9^. Five were in subjects allocated hydroxychloroquine (one of whom took no drug) and eight were in subjects allocated to no drug. So, without a single death in the three PrEP RCTs, and a highly unstable odds ratio of 0.67 for mortality in subjects allocated to hydroxychloroquine versus those who were not in the PEP RCTs (95% C.I. 0.22 to 2.05), the panel were able to conclude that this provided “ *high certainty evidence*” that hydroxychloroquine pre-exposure prophylaxis does not reduce COVID-19 mortality ^1, 2^. This is very difficult to understand, although we are told that MAGIC (the Magic Evidence Ecosystem Foundation) provided methodological support for the guidelines. For the “ *second most important outcome*” in the six RCTs there were only 49 hospital admissions in total (20 in the PrEP RCTs; 11 hydroxychloroquine, nine placebo). In the PrEP studies only two admissions were for COVID-19. These data clearly do not exclude modest but clinically significant differences in the two “ *most important*” outcomes, and most certainly do not equate to *“high certainty evidence”*.

**Figure 2.**
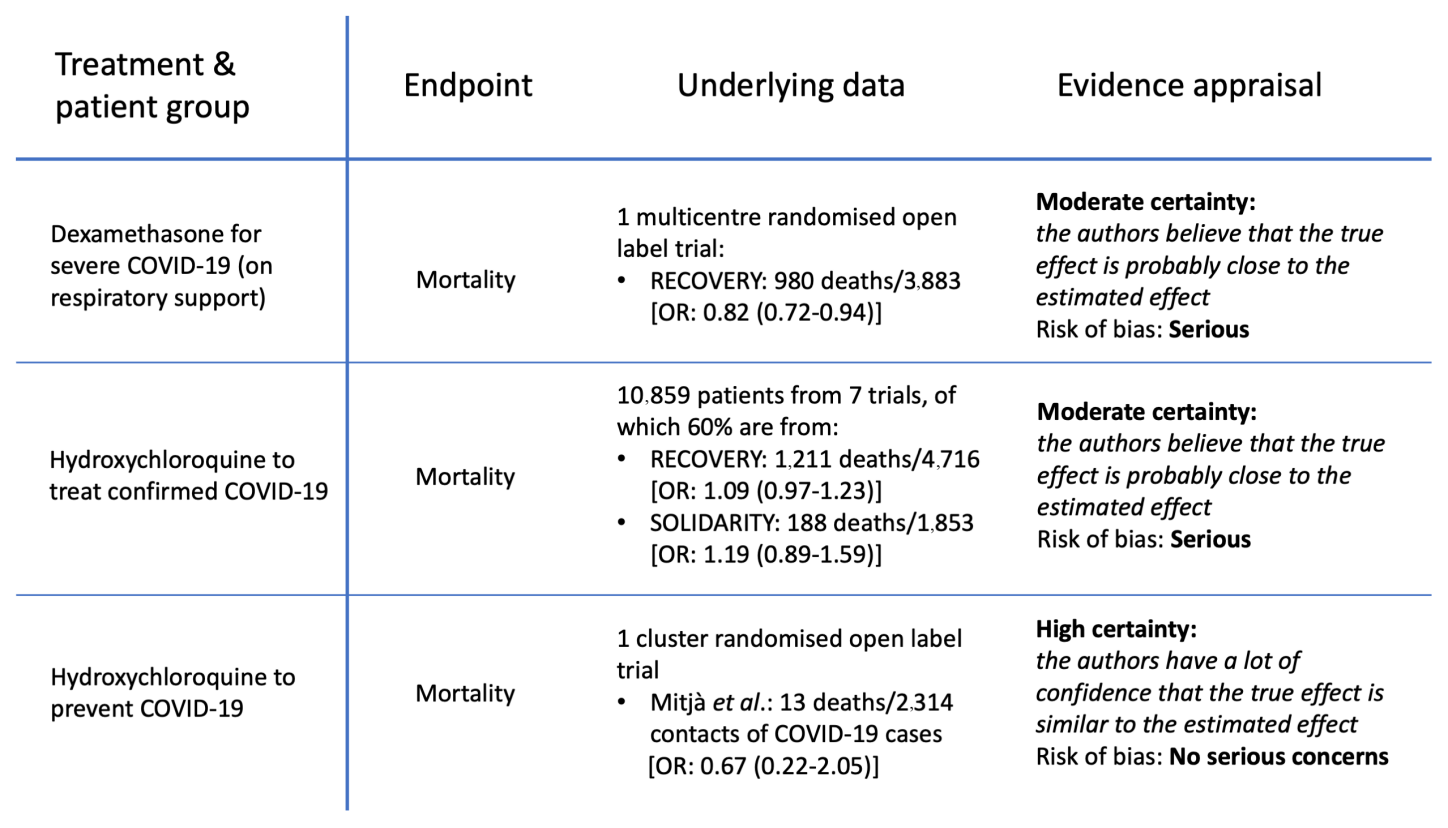
Comparison of WHO evidence grading in guidelines ^12, 13^. Upper tier: mortality outcome for dexamethasone in severe COVID-19 (hospitalised and receiving respiratory support); Middle tier: hydroxychloroquine in patients with confirmed COVID-19. This pooled data from hospitalised (87.4%) and outpatient studies (12.6%) ^14^; Lower tier: hydroxychloroquine for the prevention of COVID-19.

These evaluations should be contrasted with the earlier assessment by the WHO guideline group of dexamethasone and hydroxychloroquine in hospitalised COVID-19 patients ^11, 12^ ( Figure 2). The evidence that dexamethasone reduces mortality in hospitalised patients with COVID-19 receiving respiratory support was reviewed by the WHO guideline group in September 2020 ^11^. Their judgement was based on the very large platform RCT (RECOVERY), in which there were 980 deaths. The odds ratio for death in dexamethasone recipients receiving respiratory support was 0.82 (95%CI: 0.72 to 0.92) ^4^. For hydroxychloroquine, lack of efficacy was concluded from the outcomes of 10,859 mainly hospitalised patients (almost half from the RECOVERY trial) with over 2,000 deaths. The stratified meta-analytic estimate for mortality when combining the RECOVERY ^4^ and SOLIDARITY ^6^ trials (which used the same hydroxychloroquine dosage) resulted in a 95% confidence interval for the risk ratio of between 0.98 and 1.21 ^6^. Both of these results were graded as “ *moderate certainty evidence*” (defined as: “ *the true effect is probably close to the estimated effect*”) with serious risk of bias ( Figure 2) ^11^. So somehow these effect estimates (and thus the certainty of the treatment recommendations) based on thousands of deaths in well conducted RCTs are considered less certain (i.e. less reliable) than an estimate derived from 13 deaths.

**Figure 3.**
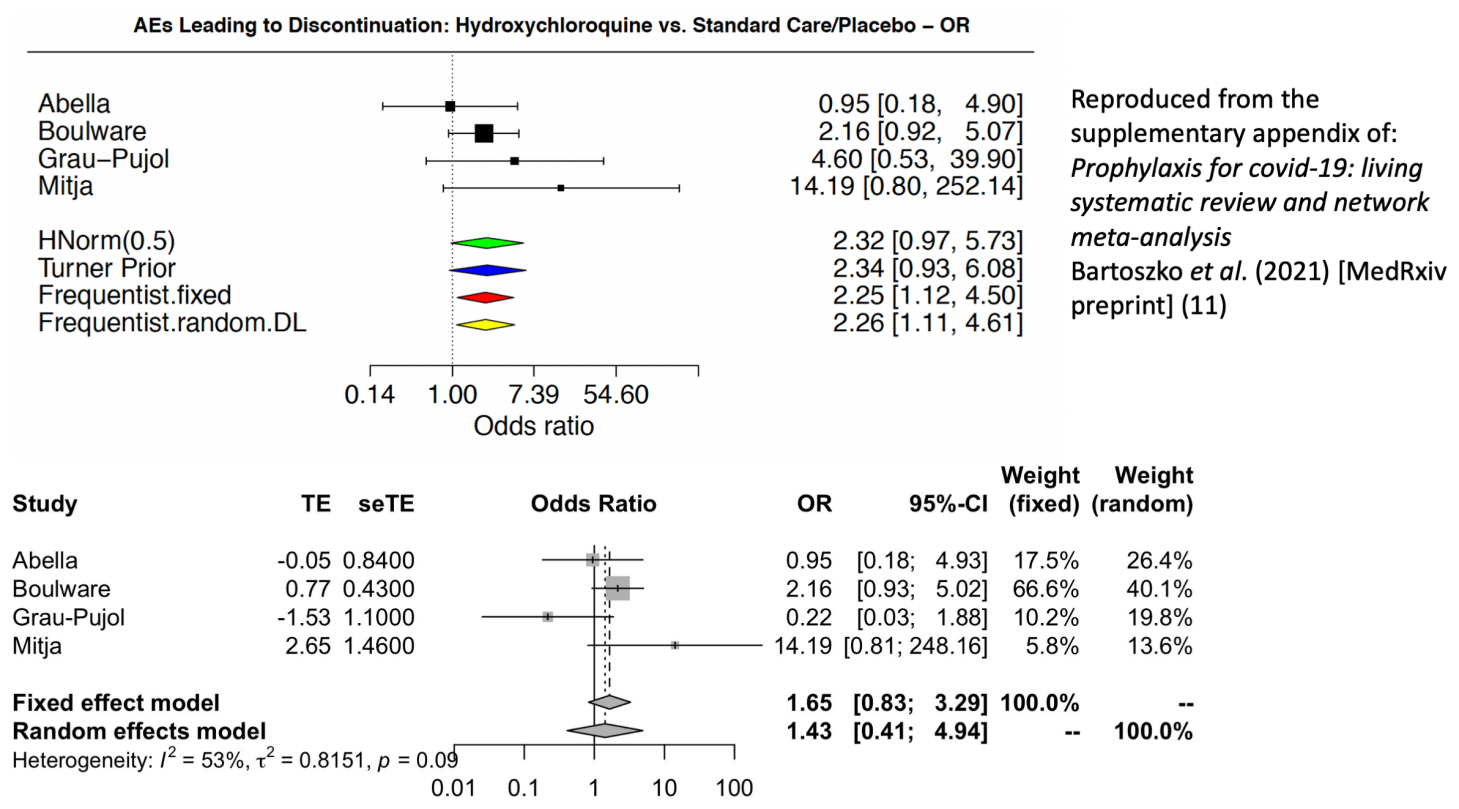
Comparison of the forest plots for adverse effects leading to discontinuation of hydroxychloroquine with the miscoding for the Grau-Pujol study (above) ^11^ and the correct coding (below).

Toxicity and tolerability are critical considerations for prophylaxis. In justifying their strong negative recommendation, the WHO guideline states that hydroxychloroquine *“probably increases the risk of adverse effects leading to discontinuation of the drug (moderate certainty)”* ^1, 2^. Aside from (i) whether it is correct to pool toxicity assessments across different dose regimens, (ii) whether the more subjective measure of discontinuation should be evaluated rather than standardised severity gradings for AEs, and (iii) whether non-placebo-controlled evidence should be included, there is an important mistake in the calculations. The WHO meta-analysis of AEs leading to study drug discontinuation miscoded the number of AE in the study by Grau-Pujol *et al*. ^8^. There were more discontinuations in the placebo group (n=5) than in the hydroxychloroquine group (n=1). Figure 3 shows the incorrect forest plot claiming a significant difference, with a corrected version below. After correction for the miscoded AEs in the Grau-Pujol study, there is no longer a significant difference and the 95% confidence interval for the odds ratio now ranges from 0.83 to 3.29. This emphasises the danger of issuing “strong” recommendations on the basis of limited and unstable data.

By issuing a judgement on the basis of scanty evidence (some of which is incorrect), and recommending that trials should stop, the WHO committee has decided that if efficacy were to be shown by continuing current trials (a 30% reduction in the risk of COVID-19 is compatible with the results from these trials ^1, 2^), then hydroxychloroquine should still not be used. What are the implications of this judgement? Any recommendation from the WHO must be taken very seriously. Such recommendations are very influential, and these may well stop all ongoing studies. Once closed, clinical trials will not reopen. More data will come from recently completed trials ^15^ but, if these are not decisive, we may never know the truth.

It is reasonable to conclude already that hydroxychloroquine does not provide high prevention or early treatment efficacies. Vaccines are rightly the priority. They give high levels of protective efficacy, and must be deployed as widely as possible. For therapeutics the preliminary evidence to date suggests that monoclonal antibodies may be more effective than small molecule repurposed antiviral candidates. But limited resource settings are unlikely to have high vaccine coverage for many months or even years, and global access to antibody therapies is very uncertain. An inexpensive, well established, widely available and relatively well tolerated drug providing moderate preventive efficacy would still be valuable- particularly in situations where there are outbreaks of vaccine escape mutations. Hydroxychloroquine is still being recommended by several countries so solid and convincing evidence of benefit or lack of benefit is still needed. The guideline group recommended that “ *resources should rather be oriented to evaluate other more promising drugs to prevent COVID-19*”. Recently registered trials are not proposing to evaluate hydroxychloroquine, so the main purpose of this recommendation seems to be to stop ongoing trials. We appreciate the urgency of COVID-19 and the need to accelerate research and share research outputs so that responsible guidance can be provided rapidly. Guidelines on the prevention and treatment of COVID-19 should be based on sufficient verified evidence, understanding of the disease process, sound statistical analysis and interpretation, and an appreciation of global needs.

## Data availability

No data are associated with this article.
