# The WHO guideline on drugs to prevent COVID-19: small numbers- big conclusions

**DOI:** 10.12688/wellcomeopenres.16741.2

**Published:** 2021-09-21

**Authors:** William HK Schilling, James J. Callery, Arjun Chandna, Raph L Hamers, James A Watson, Nicholas J White

**Affiliations:** 1 Centre for Tropical Medicine & Global Health, Nuffield Department of Medicine, University of Oxford, Oxford, OX3 7BN, UK; 2 Mahidol Oxford Tropical Medicine Research Unit (MORU), Faculty of Tropical Medicine, Mahidol University, Bangkok, Thailand; 3 Cambodia Oxford Medical Research Unit, Angkor Hospital for Children, Siem Reap, Cambodia; 4 Eijkman Oxford Clinical Research Unit, Eijkman Institute for Molecular Biology, Jakarta, Indonesia

**Keywords:** SARS-CoV-2, COVID-19, Coronavirus, Prophylaxis, Pre-exposure, Guideline

## Abstract

The World Health Organization (WHO) living guideline on drugs to prevent COVID-19 has recently advised that ongoing trials evaluating hydroxychloroquine in chemoprophylaxis should stop. The WHO guideline cites “high certainty” evidence from randomised controlled trials (RCTs) that hydroxychloroquine prophylaxis does not reduce mortality and does not reduce hospital admission, and “moderate certainty” evidence of poor tolerability because of a significantly increased rate of adverse events leading to drug discontinuation. Yet there is no such evidence. In the three pre-exposure chemoprophylaxis RCTs evaluated in the guideline there were no deaths and only two COVID-19-related hospital admissions, and there was a mistake in the analysis of the number of discontinuations (after correction there is no longer a statistically significant difference between those taking the drug and the controls). Guidelines on the prevention and treatment of COVID-19 should be based on sufficient verified evidence, understanding of the disease process, sound statistical analysis and interpretation, and an appreciation of global needs. The WHO living guideline on the prevention of COVID-19 should retract the advice to stop research on hydroxychloroquine chemoprophylaxis, should correct its errors, and should revise its guidance.

## Disclaimer

The views expressed in this article are those of the author(s). Publication in Wellcome Open Research does not imply endorsement by Wellcome.

## Introduction

World Health Organization (WHO) guidelines are generally held in high esteem because the judgements are usually based on solid and substantial evidence. However, on 2 March 2021 the WHO issued a guideline on COVID-19 chemoprophylaxis which included unusual and unjustified judgements with far reaching implications derived from small numbers of observations ^1, 2^. It also contained an important mistake. There are several analogies between the legal system and clinical investigations, and in particular the interpretation of clinical trials and the production of treatment guidelines. Both seek the truth, both review the strengths and weaknesses of evidence, and both end in a judgement. In clinical guidelines the judgement is based on an appraisal of risks and benefits. The WHO guideline uses standard methodological approaches to evaluate and grade clinical research outputs and to generate guidelines ^3^. In general, this is a conservative process requiring a substantial quality evidence base for definitive recommendations. This reduces uncertainty in the assessments, and it helps ensure that the consequent guidelines are robust. We argue that this has not happened for the WHO guideline on drugs to prevent COVID-19.

The WHO ‘living’ guideline provides a strong recommendation against hydroxychloroquine in COVID-19 pre- or post-exposure prophylaxis ^1, 2^. In many ways, hydroxychloroquine has become the COVID-19 pariah, but it still deserves a fair trial. It is true that hydroxychloroquine for both treatment and prevention was intensely politicised, and it was recommended prematurely by many governments, institutions and prominent individuals early in the COVID-19 pandemic. It should not have been. It is also true that definitive large randomised controlled trials (RCTs) have shown unequivocally that hydroxychloroquine is not life-saving in hospitalised patients ^4^. This is the stage of the disease when anti-inflammatory drugs, such as dexamethasone or interleukin 6 (IL-6) receptor antagonists, but not small molecule antiviral drugs (notably remdesivir), have proved lifesaving ^5, 6^. Unfortunately, in addition to extreme politicisation and extensive speculation, perceptions of risk from hydroxychloroquine were distorted, and ongoing trials were badly damaged by the Surgisphere® fiasco in May 2020 ^7^. A very large multinational study, led by eminent cardiologists and published in The Lancet, claimed to show that hydroxychloroquine increased the risk of lethal ventricular arrhythmias. Some regulatory authorities stopped ongoing trials immediately. However, it rapidly became apparent that the Surgisphere® data, upon which the study was based, contained implausible results, could not be accessed, and were likely fabricated. Although this paper, and another from the same team published earlier in the New England Journal of Medicine, were swiftly retracted ^7, 8^ the regulatory authorities were slow to reverse their positions, opinion swung against hydroxychloroquine despite the fraudulent report, and lasting damage was done.

It is accepted that antiviral drugs are more likely to have disease modifying efficacy early in the COVID-19 illness, whereas anti-inflammatory drugs have proven benefit later in severely ill patients. Extrapolating therapeutic results from severe disease to early illness or prevention is not justified ^9, 10^. However, in contrast to the large randomised controlled trials (RCTs) in hospitalised patients, relatively few patients have been enrolled in studies of hydroxychloroquine in early treatment, or in post-exposure (PEP) or pre-exposure prophylaxis (PrEP). In the three published, or posted, pre-exposure prophylaxis RCTs there were very few endpoints ^1, 2^. Nevertheless, the WHO guideline has concluded definitively, based upon the data from these trials, that hydroxychloroquine does not provide useful benefit in any of these situations. The WHO guideline group has also taken the unusual step of advising funders and researchers that they should reconsider the initiation and continuation of ongoing trials, i.e. they should stop. So, although it is described as a “ *living guideline*”, without further evidence it is unlikely that this particular guideline would live much longer.

From a statistical perspective both the justice system, and the institutions which issue disease prevention and treatment regulatory approvals and guidelines, focus primarily on demonstrating proof beyond reasonable doubt. Trials that lead to conviction have proved guilt beyond reasonable doubt. Pre-registration RCTs aim to prove efficacy of a drug beyond reasonable doubt, in addition to showing that the cost of this efficacy is not too high in terms of tolerability and safety. In the WHO COVID-19 prophylaxis guideline the opposite is being done. A definitive statement is made about *lack* of clinical utility. According to the guideline the highest efficacy estimate compatible with the data (lower end of the confidence interval) is not a clinically useful effect. Despite the small number of endpoints, and therefore large uncertainty, the guideline states firmly that hydroxychloroquine chemoprophylaxis results in no important differences in mortality, admission to hospital, or laboratory confirmed COVID-19 ^1, 2^. It also claims that adverse events (AEs) leading to drug discontinuation are a significant problem for hydroxychloroquine prophylaxis. Both contributed to the WHO judgement that hydroxychloroquine should not be used, and should not be evaluated further in COVID-19 prophylaxis trials ^1, 2^.

Nearly all the evidence used to generate this strong recommendation was in the public domain for several months before the March WHO guideline was published. It comprises three RCTs in post-exposure prophylaxis (PEP-which is close to early treatment) and three in pre-exposure prophylaxis (PrEP- true prevention) ^1, 2^. Two studies used confirmed or suspected COVID-19 (mainly suspected) as their primary endpoints, and the other four used laboratory-confirmed COVID-19. Dosages differed – notably, the largest PrEP study (>75% of the PrEP data) used a much lower hydroxychloroquine dose, closer to that used in antimalarial chemoprophylaxis rather than the more widely used rheumatoid arthritis doses used in other COVID-19 prophylaxis trials ^11^. Another PrEP study reported only a single case of COVID-19 ^8, 12^; and none of the data in any of the included studies were collected outside of North America or Europe. There were other differences which overall may be summarised as “substantial heterogeneity” between studies. There is certainly not enough evidence to recommend hydroxychloroquine for COVID-19 prophylaxis (there never has been), but is this small and heterogeneous evidence base enough to state conclusively, as the WHO guideline has done, that hydroxychloroquine does not provide a modest but worthwhile benefit? Does it justify the implicit recommendation that ongoing RCTs in the prevention of COVID-19 should stop now?

**Figure 1.**
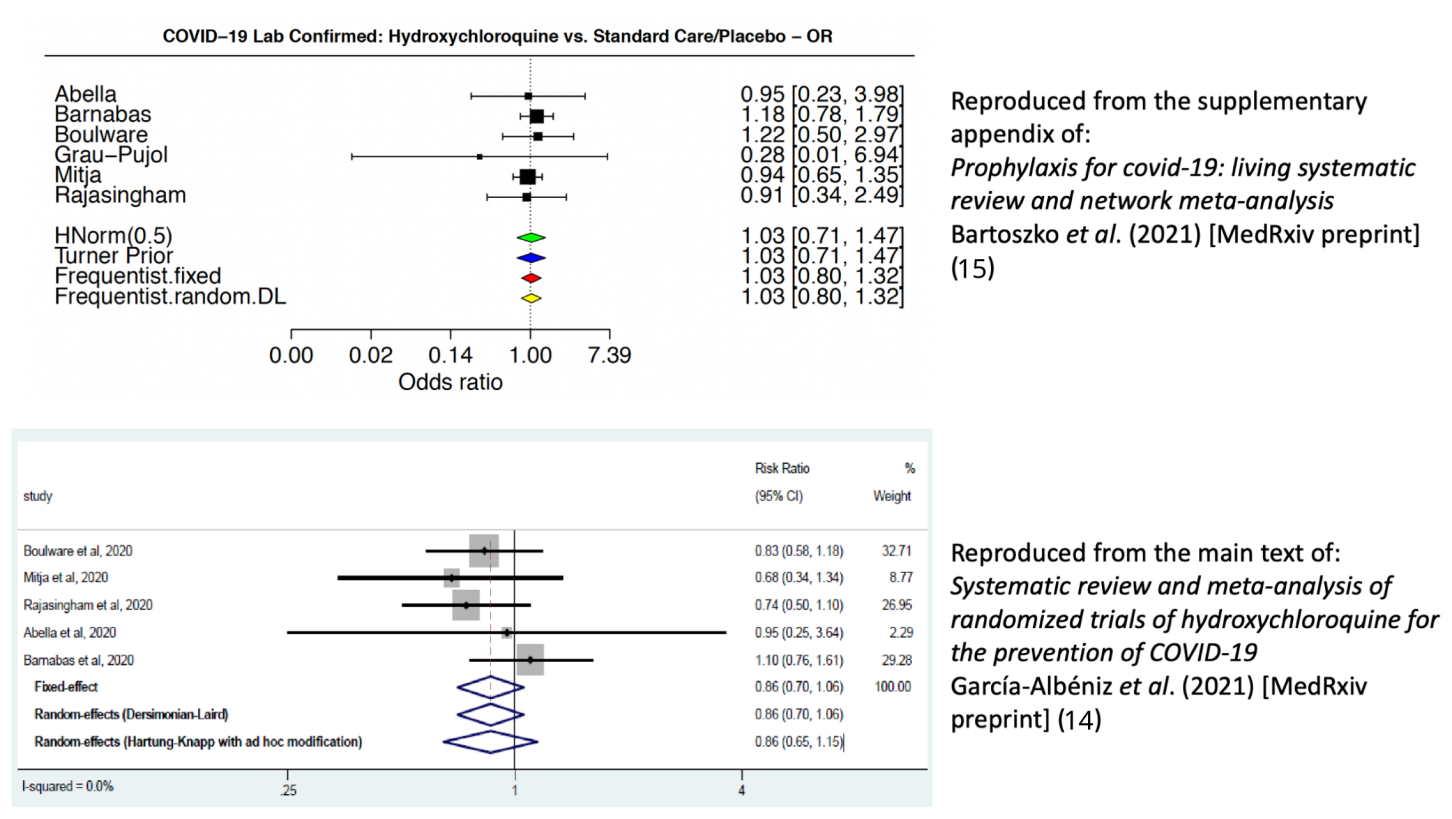
Comparison of forest plots for the effect of hydroxychloroquine in the prevention of COVID-19. The top forest plot (treatment effect summarised as odds-ratios) is reproduced from reference 15) under a CC-BY-NC 4.0 International license. This describes the evidence synthesis quoted in the WHO guidelines ^1, 2^. The forest plot used different end-points to the study primary end-points. It shows a summary odds ratio very close to 1 indicating no difference between the hydroxychloroquine and no treatment arms; the bottom forest plot (treatment effects summarised as risk ratios) is reproduced under a CC-BY-NC 4.0 International license with permission from reference 14 (main text). This does use the original study primary end-points adjusted for study design. This result is in the direction of benefit from hydroxychloroquine, although it is not statistically significant. Differences in the estimated effects between the two analyses reflect the differences in endpoint definitions and the use of intra-study adjusted treatment effects. This illustrates the sensitivity of the preventive effect estimates to the choice of end-points and methods of calculation.

These six randomised controlled comparisons enrolled 6,059 participants, but they generated few endpoints (suspected or confirmed COVID-19, hospital admission or death). In the three PrEP trials there were only 26 confirmed COVID-19 cases in total (15 of 1,197 randomised to hydroxychloroquine, 11 of 687 randomised to placebo). With so few events and considerable heterogeneity in design, the meta-analysis is sensitive to the methods employed. A previous meta-analysis chose to use the appropriately adjusted primary endpoints reported in each hydroxychloroquine prevention study (e.g. one study was a cluster randomised trial ^13^ so adjustment for cluster was necessary) ^14^. This estimated a meta-analytic risk ratio of 0.86 (95% confidence interval [C.I.] 0.70 to 1.06) in the direction of benefit from hydroxychloroquine. In comparison, the WHO guideline decided to use laboratory confirmed COVID-19 (asymptomatic and symptomatic) for the primary endpoint in their meta-analysis of virological effect, without intra-study adjustments, resulting in a meta-analytic odds ratio of 1.03 (95% C.I. 0.80 to 1.32) ^1, 2^. Figure 1 compares the two analyses.

The WHO guideline development panel decided that “ *Mortality would be the outcome most important to individuals, followed by need for hospital admission, laboratory confirmed SARS-CoV-2 infection, and adverse effects leading to discontinuation*” ^1, 2^. The guideline review determined that there was no important difference in mortality, admission to hospital, or laboratory confirmed COVID-19, and that the evidence quality to support these statements was high. The four-star “ *High GRADE rating*” is defined as: “ *the authors have a lot of confidence that the true effect is similar to the estimated effect*”. Yet there were only 13 deaths in total in the six prophylaxis trials, and all 13 were from one cluster-randomised non-blinded PEP trial ^13^. Five were in subjects allocated hydroxychloroquine (one of whom took no drug) and eight were in subjects allocated to no drug. So, without a single death in the three PrEP RCTs, and a highly unstable odds ratio of 0.67 for a mortality benefit in subjects allocated to hydroxychloroquine versus those who were not in the PEP RCTs (95% C.I. 0.22 to 2.05), the WHO panel were able to conclude that this provided “ *high certainty evidence*” that hydroxychloroquine pre-exposure prophylaxis does not reduce COVID-19 mortality ^1, 2^. This is very difficult to understand, although we are told that MAGIC (the Magic Evidence Ecosystem Foundation) provided methodological support for the guidelines. For the “ *second most important outcome*” in the six RCTs there were only 49 hospital admissions in total (20 in the PrEP RCTs; 11 hydroxychloroquine, nine placebo). In the PrEP studies only two admissions were for COVID-19. These data clearly do not exclude modest but clinically significant differences in the two “ *most important*” outcomes, and most certainly do not equate to *“high certainty evidence”*.

**Figure 2.**
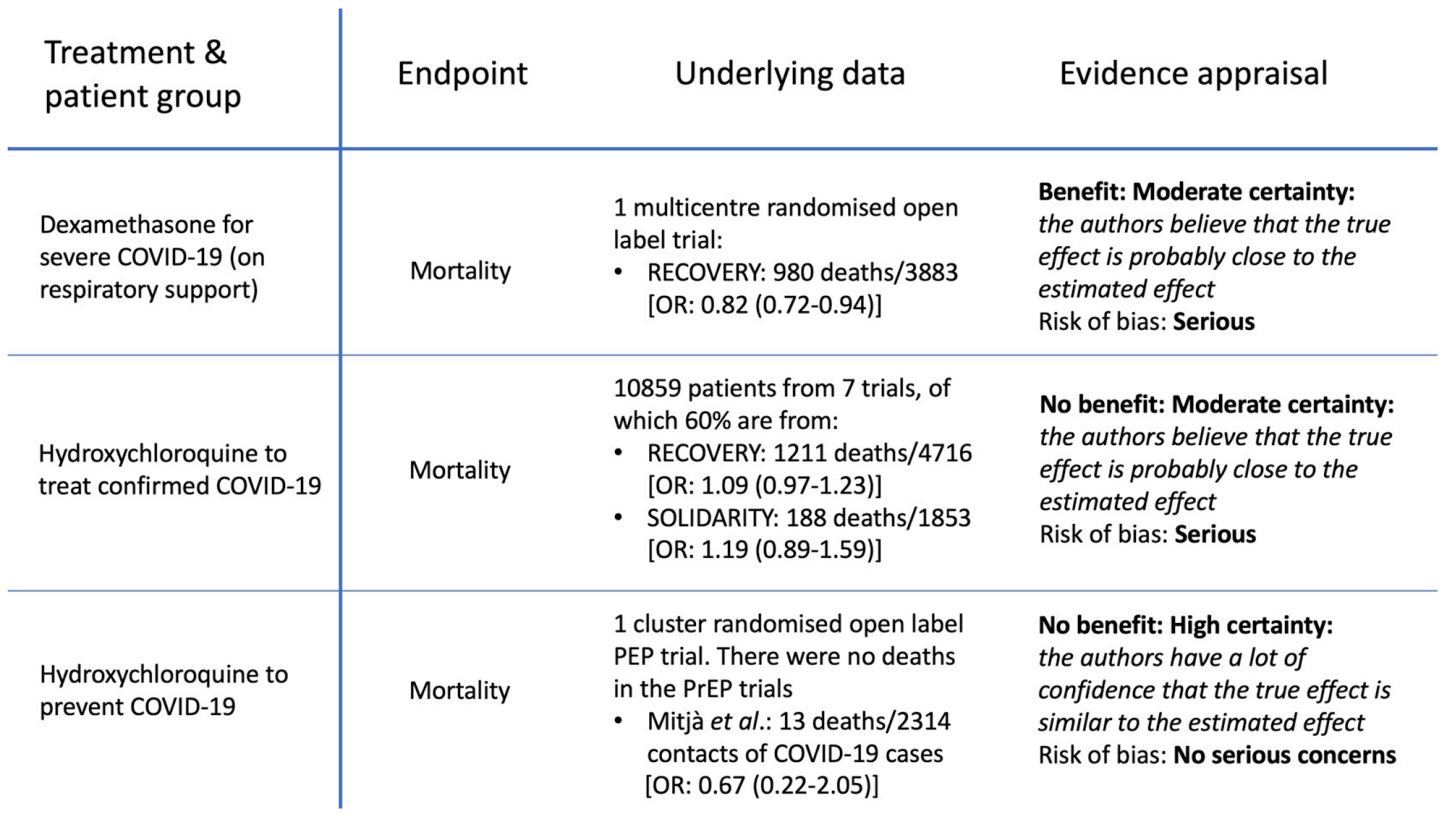
Comparison of WHO evidence grading in guidelines ^16, 17^. Upper tier: mortality outcome for dexamethasone in severe COVID-19 (hospitalised and receiving respiratory support); Middle tier: hydroxychloroquine in patients with confirmed COVID-19. This pooled data from hospitalised (87.4%) and outpatient studies (12.6%) ^14^; Lower tier: hydroxychloroquine for the prevention of COVID-19 ^1, 2^.

These chemoprevention evaluations ^1, 2, 15^ should be contrasted with the earlier treatment assessments by the WHO guideline group of dexamethasone and hydroxychloroquine in hospitalised COVID-19 patients ^16, 17^ ( Figure 2). The evidence that dexamethasone reduces mortality in hospitalised patients with COVID-19 receiving respiratory support was reviewed by the WHO guideline group in September 2020 ^16^. Their judgement was based on the very large platform RCT (RECOVERY), in which there were 980 deaths. The odds ratio for death in dexamethasone recipients receiving respiratory support was 0.82 (95%CI: 0.72 to 0.92) ^4^. As a result, dexamethasone has since become universally adopted as standard of care for hospitalised patients with COVID-19 who require respiratory support. For hydroxychloroquine, lack of efficacy was concluded from the outcomes of 10,859 mainly hospitalised patients (almost half from the RECOVERY trial) with over 2,000 deaths. The stratified meta-analytic estimate for mortality when combining the RECOVERY ^4^ and SOLIDARITY ^6^ trials (which used the same hydroxychloroquine dosage) resulted in a 95% confidence interval for the risk ratio of between 0.98 and 1.21 ^6^. However, both of these results were graded only as “ *moderate certainty evidence*” (defined as: “ *the true effect is probably close to the estimated effect*”) with serious risk of bias ( Figure 2) ^16, 17^. So somehow these effect estimates (and thus the certainty of the treatment recommendations) based on thousands of deaths in well conducted RCTs are considered less certain (i.e. less reliable) than an estimate derived from 13 deaths (5 versus 8) in a single study.

**Figure 3.**
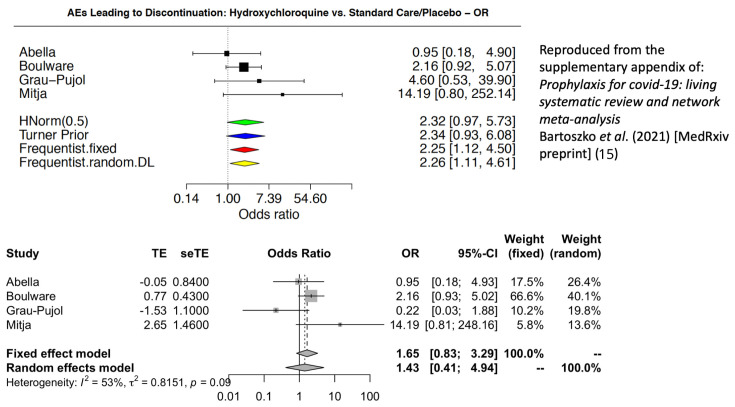
Comparison of the forest plots for adverse effects leading to discontinuation of hydroxychloroquine ^15^ with the miscoding for the Grau-Pujol study (above) ^12^ and the correct coding (below).

Toxicity and tolerability are critical considerations for prophylaxis. In justifying their strong negative recommendation, the WHO chemoprevention guideline states that hydroxychloroquine *“probably increases the risk of adverse effects leading to discontinuation of the drug (moderate certainty)”* ^1, 2^. Aside from (i) whether it is correct to pool toxicity assessments across different dose regimens, (ii) whether the more subjective measure of discontinuation should be evaluated rather than standardised severity gradings for AEs, and (iii) whether unblinded evidence should be included, there is an important mistake in the calculations. The WHO meta-analysis of AEs leading to study drug discontinuation miscoded the number of AEs in the study by Grau-Pujol *et al*. ^12^. There were actually more discontinuations in the placebo group (n=5) than in the hydroxychloroquine group (n=1) (the WHO analysis had these figures mistakenly reversed). Figure 3 shows the incorrect forest plot claiming a significant difference, with a corrected version below. After correction for the miscoded AEs in the Grau-Pujol study, there is no longer a significant difference and the 95% confidence interval for the odds ratio now ranges from 0.83 to 3.29. This emphasises the danger of issuing “strong” recommendations on the basis of unverified and limited data.

By issuing a strong judgement on the basis of scanty evidence (some of which is incorrect), and recommending that trials should stop, the WHO committee has decided that if efficacy were to be shown by continuing current trials (a 30% reduction in the risk of COVID-19 is compatible with the results from these trials ^1, 2^), then hydroxychloroquine should still not be used. What are the implications of this judgement? Any recommendation from the WHO must be taken very seriously. Such recommendations are very influential, and these may well stop all ongoing studies. Once closed, clinical trials will not reopen. If followed, this recommendation could prevent us ever knowing the truth.

It is reasonable to conclude already that hydroxychloroquine does not provide high prevention or early treatment efficacies. Vaccines are rightly the priority. The COVID-19 vaccines give high levels of protective efficacy, albeit of limited duration, and must be deployed as widely as possible. For therapeutics the preliminary evidence to date suggests that monoclonal antibodies are more effective than small molecule repurposed antiviral candidates ^18^. But limited resource settings are unlikely to have high vaccine coverage for many months or even years, and global access to expensive antibody therapies is very uncertain. An inexpensive, well established, widely available and relatively well tolerated drug providing moderate preventive efficacy would still be valuable- particularly in situations where there are outbreaks of vaccine escape mutations. Hydroxychloroquine is still being recommended by several countries so solid and convincing evidence of benefit or lack of benefit is still needed. The guideline group recommended that “ *resources should rather be oriented to evaluate other more promising drugs to prevent COVID-19*”. Recently registered trials are not proposing to evaluate hydroxychloroquine, so the main purpose of this recommendation seems to be to stop ongoing trials. We appreciate the urgency of COVID-19 and the need to accelerate research and share research outputs so that responsible guidance can be provided rapidly. The risks and the benefits of hydroxychloroquine and chloroquine COVID-19 prophylaxis have not yet been characterised adequately. The WHO guideline is based on inadequate evidence and erroneous judgements. Randomised controlled trials should continue. Guidelines on the prevention and treatment of COVID-19 should be based on sufficient verified evidence, understanding of the disease process, sound statistical analysis and interpretation, and an appreciation of global needs.

## Updates from September 2021

Since this paper was submitted originally in March, both the BMJ ( https://www.bmj.com/content/372/bmj.n526/rapid-responses) and the authors of the WHO guideline were contacted in April and the information above was provided to them. However, the WHO living guideline has remained unchanged, and the error in the adverse events still remains uncorrected. In May a large open-label cluster-randomised controlled trial from Singapore was reported which evaluated five different chemoprophylaxis regimens over a six-week period ^19^. This trial had substantially more confirmed SARS CoV-2 infections than all the other chemoprevention trials combined. The proportion of patients in the hydroxychloroquine arm who developed laboratory confirmed COVID-19 (212/432; 49%) was significantly lower than in the reference (Vitamin C) arm (433/619; 70%); p=0.01. Hydroxychloroquine (400 mg salt loading dose followed by 200 mg/day) chemoprophylaxis was very well tolerated. Results of a double-blind placebo-controlled trial of hydroxychloroquine prophylaxis (200mg/day) from Mexico posted as a preprint ^20^, reported that 1/62 subjects assigned to hydroxychloroquine and 6/65 randomised to placebo developed COVID-19. (Log Rank test p = 0.09). Results of another, larger, hydroxychloroquine prophylaxis placebo-controlled RCT (400mg/day) from the USA (HERO-HCQ) were also recently published as a preprint. 41/683 (6.0%) in the hydroxychloroquine arm and 53/676 (7.8%) in the placebo arm met the primary clinical end-point of confirmed or suspected COVID-19. The difference was not statistically significant but again there were no safety issues ^21^. Taken together these recently reported results provide additional confirmation of the safety and tolerability of hydroxychloroquine COVID-19 chemoprophylaxis and, while being far from conclusive, they point further in the direction of benefit in preventing the infection. This reinforces the arguments above in the main paper that the WHO guideline judgement is unjustifiable based on the presented evidence, and it emphasizes the need to continue rather than stop obtaining more information to provide evidence-based guidance.

## Data availability

No data are associated with this article.
