# Supplementary material for: The WHO guideline on drugs to prevent COVID-19: small numbers- big conclusions
Source: Wellcome Open Res. 2021 Sep 21;6:71. Originally published 2021 Mar 30. [Version 2] doi: 10.12688/wellcomeopenres.16741.2 (PMC8356259; doi:10.12688/wellcomeopenres.16741.2)
Supplement: Peer review history

# Peer review: Reviewer response for version 1

**DOI:** 10.21956/wellcomeopenres.18462.r44989

**Published:** 2021-08-09

**Authors:** Li Yang Hsu

**Affiliations:** 1 Saw Swee Hock School of Public Health, National University of Singapore, Singapore, Singapore

The authors have put forth a cogent argument that the WHO guideline lacked sufficiently strong evidence to support the positions that it had taken. They have, however, also acknowledged that hydroxychloroquine (HCQ) does not provide high prevention or post-exposure efficacy. Based on the trials published/ongoing, it might be possible that HCQ might yet be found to provide "moderate efficacy" in prophylaxis against COVID-19. It is equally if not more likely that the results would demonstrate lack of significant efficacy, while subjecting the participants to a small but definite risk of harm.

 I think it might be helpful if the authors clarified their position at their end. Should all ongoing HCQ  prophylaxis trials be continued, or should there be some attempt at coordination so that only one or a minimum number of definitive trials be run? Taken to the extreme, this could mean that trials could continue to be run for different dosages of HCQ, or in combination with other agents. The issue of focusing resources is an important one, particularly during this ongoing pandemic.

 Finally, I could not find in the letter any statement on whether some or all authors are involved in HCQ prophylaxis trials. It might be helpful for purposes of transparency to state this.

Does the article adequately reference differing views and opinions?

Yes

Are all factual statements correct, and are statements and arguments made adequately supported by citations?

Yes

Is the Open Letter written in accessible language?

Yes

Where applicable, are recommendations and next steps explained clearly for others to follow?

Yes

Is the rationale for the Open Letter provided in sufficient detail?

Partly

Reviewer Expertise:

Infectious diseases

I confirm that I have read this submission and believe that I have an appropriate level of expertise to confirm that it is of an acceptable scientific standard.

# Peer review: Reviewer response for version 1

**DOI:** 10.21956/wellcomeopenres.18462.r43685

**Published:** 2021-05-24

**Authors:** Paul Anantharajah Tambyah

**Affiliations:** 1 Department of Medicine, Yong Loo Lin School of Medicine, National University of Singapore, Singapore, Singapore

This is a very important open letter that raises significant questions about a "strong" recommendation from the WHO living guideline. The points are made well but there are some areas which could be clearer.

1. The analogy with a legal judgement is good but there are differences. Most decisions in clinical medicine are not as clear cut as "guilty or not guilty" and they offer a balance of risks vs benefits. This is important especially in this case as the risks have been significantly distorted by a retracted paper which had a material effect on recruitment on some of the larger studies cited.
2. In a similar vein, it would have been good to have a couple of lines at least about the Surgisphere fiasco and its impact on recruitment for major ongoing clinical trials at the time. While the fiasco is still fresh in the minds of many of us now, it will be long forgotten before some time and it would be good to have a critical record of the impact of the rush to publication by some of the major medical journals who may not have exercised the same degree of due diligence that would have been exercised pre-pandemic.
3. While the forest plots are interesting especially to statisticians, for most clinicians, they are not that easy to understand and by separating them, it is easy to misunderstand whether the authors of this open letter are endorsing the views of the WHO guideline authors.
4. It would be good to have a table listing the trials that were included in the WHO guideline and to list the strengths and weaknesses of each study (e.g. sample size, end point, etc.).
5. It would also be interesting to point out the direction and magnitude of the impact of HCQ which seems to be quite consistent in the studies. Small but not negative and similarly for the adverse effect profile.
6. It would also be good to add in the studies which are still ongoing or have been published recently - perhaps as a supplementary appendix if this is done with this journal.

Does the article adequately reference differing views and opinions?

Partly

Are all factual statements correct, and are statements and arguments made adequately supported by citations?

Yes

Is the Open Letter written in accessible language?

Yes

Where applicable, are recommendations and next steps explained clearly for others to follow?

Partly

Is the rationale for the Open Letter provided in sufficient detail?

Yes

Reviewer Expertise:

Clinical trials, emerging infectious diseases

I confirm that I have read this submission and believe that I have an appropriate level of expertise to confirm that it is of an acceptable scientific standard.
